# Supplementary material for: The Inverted U-Shaped Association between Serum Vitamin D and Serum Uric Acid Status in Children and Adolescents: A Large Cross-Sectional and Longitudinal Analysis
Source: Nutrients. 2024 May 15;16(10):1492. doi: 10.3390/nu16101492 (PMC11124299; doi:10.3390/nu16101492)
Supplement: Supplementary file 1 [file nutrients-16-01492-s001.zip › nutrients-2977169-supplementary.pdf]

The inverted U-shaped association between serum vitamin D and serum uric acid status in children and adolescents: a large cross-sectional and longitudinal analysis

Supplementary online content

Figure S1. Flow chart of study design with participant enrollment.

Table S1. The follow-up characteristics of 1641 participants by quartiles of serum 25(OH)D.

Table S2. Stratified analyses of potential modification effect for the association between serum 25(OH)D and SUA.

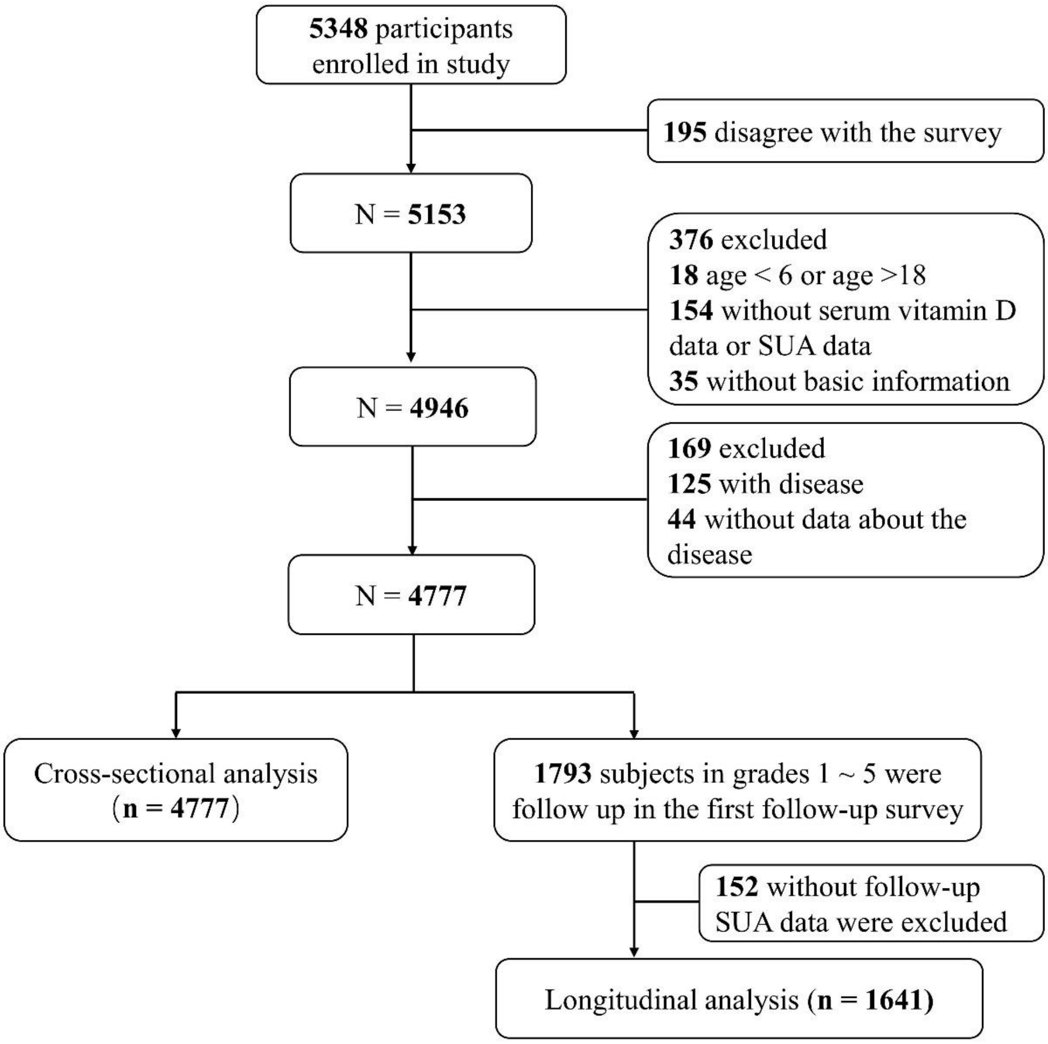

Figure S1. Flow chart of study design with participant enrollment

Table S1. The follow-up characteristics of 1641 participants by quartiles of serum 25(OH)D.

	Total	Q1	Q2	Q3	Q4	P-value
**N**	1641	411	412	408	410	
**Age, years**	10.33[9.04,11.53]	10.83[9.27,11.92]	10.60[9.27,11.64]	10.21[8.91,11.24]	9.85[8.76,11.07]	<0.001
**Female, n (%)**	738(45.0)	181(44.0)	232(56.3)	240(58.8)	250(61.0)	<0.001
**Waist, cm**	55.00 [51.00, 61.00]	55.00 [51.30, 61.00]	55.00 [51.00, 62.52]	54.50 [51.00, 60.00]	54.50 [50.50, 59.25]	0.126
**BMI, kg/m2**	15.82[14.50, 17.71]	15.87 [14.62, 18.06]	15.91 [14.52, 17.97]	15.75 [14.50, 17.50]	15.75 [14.40, 17.48]	0.268
**Weight status, n (%)**						0.684
Underweight	194(11.8)	48(11.7)	51(12.4)	43(10.5)	52(12.7)	
Normal	1106(67.4)	285(69.3)	276(67.0)	274(67.2)	271(66.1)	
Overweight	181(11.0)	35(8.5)	45(10.9)	48(11.8)	53(12.9)	
Obesity	160(9.8)	43(10.5)	40(9.7)	43(10.5)	34(8.3)	
**Puberty stage, n (%)**						<0.001
Pre-puberty	1166(71.1)	251(61.1)	278(67.5)	307(75.2)	330(80.5)	
Mid-puberty	416(25.4)	126(30.7)	122(29.6)	97(23.8)	71(17.3)	
Post-puberty	59(3.6)	34(8.3)	12(2.9)	4(1.0)	9(2.2)	
**Household income, n (%)**						0.003
< 120 000 CNY per year	394(24.0)	122(29.7)	102(24.8)	91(22.3)	79(19.3)	
~250 000 CNY per year	538(32.8)	145(35.3)	133(32.3)	122(29.9)	138(33.7)	
≥250 000 CNY per year	696(42.4)	141(34.3)	175(42.5)	189(46.3)	191(46.6)	
Missing	13(0.8)	3(0.7)	2(0.5)	6(1.5)	2(0.5)	
**Paternal education level, n (%)**						0.002
≤ 9years	170(10.4)	53(12.9)	43(10.4)	39(9.6)	35(8.5)	
~12years	347(21.1)	112(27.3)	88(21.4)	78(19.1)	69(16.8)	
~15years	462(28.2)	119(29.0)	102(24.8)	120(29.4)	121(29.5)	
≥ 16years	654(39.9)	125(30.4)	177(43.0)	169(41.4)	183(44.6)	
Missing	8(0.5)	2(0.5)	2(0.5)	2(0.5)	2(0.5)	
**Maternal education level, n (%)**						0.002
≤ 9years	233(14.2)	65(15.8)	65(15.8)	54(13.2)	49(12.0)	
~12years	365(22.2)	122(29.7)	82(19.9)	82(20.1)	79(19.3)	
~15years	477(29.1)	96(23.4)	111(26.9)	138(33.8)	132(32.2)	
≥ 16years	552(33.6)	125(30.4)	151(36.7)	131(32.1)	145(35.4)	
Missing	14(0.9)	3(0.7)	3(0.7)	3(0.7)	5(1.2)	
**Vitamin D supplement, n (%)**						<0.001
No	1373(83.7)	369(89.8)	337(81.8)	341(83.6)	326(79.5)	
Yes	265(16.1)	42(10.2)	72(17.5)	67(16.4)	84(20.5)	
Missing	3(0.2)	0(0.0)	3(0.7)	0(0.0)	0(0.0)	
**Multivitamin mineral supplement, n (%)**						0.391
No	1497(91.2)	370(90.0)	374(90.8)	384(94.1)	369(90.0)	
Yes	134(8.2)	39(9.5)	35(8.5)	22(5.4)	38(9.3)	
Missing	10(0.6)	2(0.5)	3(0.7)	2(0.5)	3(0.7)	
**Smoking status, n (%)**						0.651
Never	1609(98.0)	402(97.8)	404(98.1)	404(99.0)	399(97.3)	
< 1cigarette per month	8(0.5)	3(0.7)	2(0.5)	1(0.2)	2(0.5)	
≥ 1cigarette per month	2(0.1)	0(0.0)	0(0.0)	1(0.2)	1(0.2)	
e-Cigarette	1(0.1)	0(0.0)	1(0.2)	0(0.0)	0(0.0)	
Missing	21(1.3)	6(1.5)	5(1.2)	2(0.5)	8(2.0)	
**Drinking status, n (%)**						0.429
Never	1588(96.8)	396(96.4)	396(96.1)	401(98.3)	395(96.3)	
< 1standard drink per month	43(2.6)	13(3.2)	11(2.7)	6(1.5)	13(3.2)	
≥ 1standard drink per month	2(0.1)	0(0.0)	1(0.2)	1(0.2)	0(0.0)	
Missing	8(0.5)	2(0.5)	4(1.0)	0(0.0)	2(0.5)	
**Levels of MVPA, n (%)**						0.315
< 0.5hourperd	478(29.1)	123(29.9)	112(27.2)	122(29.9)	121(29.5)	
~1hourperd	725(44.2)	171(41.6)	189(45.9)	177(43.4)	188(45.9)	
~3hoursperd	374(22.8)	98(23.8)	91(22.1)	90(22.1)	95(23.2)	
> 3hoursperd	49(3.0)	17(4.1)	15(3.6)	13(3.2)	4(1.0)	
Missing	15(0.9)	2(0.5)	5(1.2)	6(1.5)	2(0.5)	
**SUA-annual variation, μmol/(L*year)**	24.40 [20.35, 28.65]	25.01[-4.61, 51.37]	19.85[-5.62, 48.58]	15.15[-10.56, 43.05]	17.08[-7.70, 44.14]	0.047
**25(OH)D, ng/mL**	0.39 [0.26, 0.57]	18.12[16.18, 19.34]	22.42[21.41, 23.45]	26.17[25.28, 27.35]	31.77[29.89, 34.48]	<0.001
**25(OH)D_2, ng/mL**	23.80 [19.79, 28.14]	0.36[0.24,0.53]	0.39[0.26,0.59]	0.42[0.28,0.64]	0.40[0.28,0.57]	<0.001
**25(OH)D_3, ng/mL**	15.17 [13.75, 16.04]	17.69[15.68, 18.86]	21.91[20.94, 22.92]	25.66[24.67, 26.75]	31.34[29.46, 33.84]	<0.001

Abbreviations: BMI, body mass index; CNY, Chinese Yuan; MVPA, moderate to vigorous physical activity; SUA, serum uric acid.

Table S2. Stratified analyses of potential modification effect for the association between serum 25(OH)D and SUA.

		Concentrations < 24.31 ng/mL			Concentrations ≥ 24.31 ng/mL	
	N	Adjusted β coefficient (95% CI)	P for interaction	N	Adjusted β coefficient (95% CI)	*P* for interaction
**Sex**			< 0.0001			0.0901
Male	1617	5.97(-5.82, 17.77)		1035	-6.63(-17.73, 4.48)	
Female	1542	6.43(-2.77, 15.64)		583	1.26(-13.20, 15.73)	
**Age**			0.0008			0.1491
6-12	1565	-4.09(-14.86, 6.69)		1273	-9.12(-18.38, 0.13)	
12-18	1594	5.62(-4.74, 15.98)		345	17.24(-8.43, 42.91)	
**Stage of puberty**			0.0332			0.4826
Pre-puberty	662	3.13(-12.64, 18.90)		832	-1.01(-10.81, 8.78)	
Mid-puberty	663	8.15(-10.23, 26.52)		397	-15.21(-36.57, 6.16)	
Post-puberty	1834	7.58(-1.88, 17.03)		389	10.09(-12.97, 33.15)	
**Weight status**			0.8208			0.1970
Underweight	250	-0.69(-28.82, 27.44)		170	-3.61(-28.82, 27.44)	
Normal	2198	6.66(-2.20, 15.53)		1072	-4.19(-14.85, 6.47)	
Overweight	711	8.11(-10.47, 26.70)		376	-11.73(-34.21, 10.75)	
**Central obesity**			0.3447			0.3852
Yes	433	6.30(-16.68, 29.28)		193	-29.11(-65.86, 7.65)	
No	2613	10.21(1.96, 18.46)		1254	-3.72(-13.72, 6.28)	

Generalized linear models were used. Model adjusted for age, gender, weight status, pubertal development stages, household income, parental education levels, maternal education levels, smoking and drinking status, vitamin D supplement, multivitamin mineral supplement, levels of MVPA.
P value for interaction was obtained by using the Wald test.
SUA corresponds to units of μmol/L.
